# Low-MESF fluorescent reference particles for flow cytometry analysis of extracellular vesicles

**DOI:** 10.1038/s41598-026-60804-3

**Published:** 2026-09-01

**Authors:** Marcell Pálmai, Viet Thuc Dang, Anikó Gaál, Kinga Ilyés, István Mándity, Judith Mihály, Andy I. Nguyen, Preston T. Snee, Zoltán Varga

**Affiliations:** 1 Institute of Materials and Environmental Chemistry, HUN-REN Research Centre for Natural Sciences, Budapest, Hungary; 2 Department of Chemistry, University of Illinois Chicago, Chicago, IL USA; 3 Hevesy György PhD School of Chemistry, Eötvös Loránd University, Budapest, Hungary; 4 Department of Physical Chemistry and Materials Science, Budapest University of Technology and Economics, Budapest, Hungary; 5 Department of Chemistry, Eszterházy Károly Catholic University, Eger, Hungary; 6 Institute of Organic Chemistry, Semmelweis University, Budapest, Hungary

**Keywords:** Flow cytometry, Extracellular vesicles, Fluorescence calibration, R5 peptide, Silica particles, Biological techniques, Biotechnology, Chemistry, Nanoscience and technology

## Abstract

Extracellular vesicles (EVs) hold strong potential as non-invasive biomarkers for early detection of diseases. However, their clinically relevant quantitative flow cytometric (FCM) analysis remains limited by the lack of suitable calibration particles with low single-particle fluorescence values. Although fluorescent labeling of silica particles is well established, traditional approaches typically yield heterogeneous fluorophore distributions in the low MESF (Molecules of equivalent soluble fluorophore) range, which is required for single-EV fluorescence calibration. To this end, we introduce a labeling strategy that overcomes this limitation by using fluorescently tagged silica-binding peptides (R5-FITC) to produce submicron silica particles with homogeneous low-level fluorophore coverage. Silica particles of three different sizes were synthesized and labeled in a fast and straightforward manner. After comprehensive characterization, FCM investigations revealed that the resulting dim fluorescent silica particles (as low as 278 MESF with narrow distribution) are suitable for calibrating the fluorescence intensity of EVs isolated from red blood cells. Our findings show that fluorescent particles resembling EVs in both size and fluorescence can improve calibration accuracy and foster standardization in FCM-based EV characterization.

**Supplementary Information:**

The online version contains supplementary material available at https://doi.org/10.1038/s41598-026-60804-3.

## Introduction

Extracellular vesicles (EVs) are small cell-derived particles that play an important role in intercellular communication^1,2^. They carry proteins and nucleic acids, surrounded by a phospholipid bilayer, and contribute to many physiological and pathological processes^3,4^. For example, immune cells can release antigen-presenting EVs^5^, tumor cells have been shown to exploit EVs to contribute to their progression and suppress the immune system^6,7^, EVs participate in neuronal survival^8^, and β-amyloid peptides propagate to other cells by EVs^9,10^. Given their presence in all body fluids, they have vast potential as non-invasive or minimally invasive biomarkers for the early detection of abnormalities and health conditions^11–15^.

Although the field of EVs is expanding rapidly, they have not yet been widely translated into clinical practice mainly due to their biophysical, biochemical, and functional heterogeneity^16^. This heterogeneity arises from the diverse types and functional states of the releasing cells, as well as the different biogenesis pathways. Based on their biogenesis EVs can be broadly grouped into two types: exosomes, which originate from the endosomal pathway, and ectosomes which bud directly from the plasma membrane^5^. EVs can also be classified into several types based on their size. Small EVs (e.g., exosomes), with an approximate diameter of 50 to 200 nm, are the most abundant in biological fluids. Medium-sized EVs (e.g., microvesicles), ranging from 200 to 800 nm in diameter, are less numerous, while large EVs (≥ 800 nm; e.g., apoptotic bodies) are the least abundant^5^. Such heterogeneity in composition, size and concentration presents unresolved difficulties in detecting, classifying, and characterizing them. Furthermore, the absence of established reference concentration ranges and standardization impedes the comparability of results reported by different research groups^17^. In other words, EV measurement results cannot be compared because those measurements are not traceable to international standards.

Various techniques have been utilized for size characterization and phenotyping of EVs^18–20^ including confocal microscopy, dynamic light scattering (DLS), infrared spectroscopy (IR), nanoparticle tracking analysis (NTA), resistive pulse sensing, transmission electron microscopy (TEM), and flow cytometry (FCM), among others. FCM detects particles based on light scattering and fluorescence and is widely used in both clinical diagnostics and basic research. It is a high-throughput, single-particle analysis technique that can analyze thousands of particles per second, making it highly efficient for large numbers of samples. Compared to TEM, NTA, DLS, and biochemical assays that are often limited to specific aspects of EV analysis (e.g., size, concentration, or specific protein detection), FCM provides a more comprehensive approach by combining size, concentration, and fluorescence-based phenotyping in a single analysis.

One issue with FCM concerns the fact that light scattering and fluorescence intensities are reported in arbitrary units, which poses a challenge in accurately determining the size of EVs and the number of antigens on individual vesicles. Standardization is essential to facilitate the comparison of EV measurements across FCM laboratories^21^. The pivotal factor in achieving this standardization lies in calibration, which requires reference particles that approximate the light scattering and fluorescence properties of EVs (labeled with fluorescent antibodies) and can convert measured scattering and fluorescence values from arbitrary units to absolute units. Calibrating the scattering and fluorescence intensities enables the determination of the size of individual particles and quantification of the fluorescence of individual particles in terms of MESF (Molecules of Equivalent Soluble Fluorophore) or ERF (Equivalent Reference Fluorophore) units, respectively.

Within quantitative FCM, a well-established method for fluorescence calibration of flow cytometers involves a set of reference microparticles labeled with defined quantities of fluorophores, allowing fluorescence intensity to be quantified in standardized MESF units^22–24^. However, these microparticles were developed for the FCM analysis of cells; therefore, they have much higher MESF values than typical EVs labeled with fluorescent antibodies. And even though particles with smaller sizes and lower MESF values (e.g., the 0.1–0.5 μm ViroCheck NanoParticle Reference Kit by Invitrogen^™^ and the 0.4–0.6 μm SPHERO™ Rainbow Calibration Particles by Spherotech) have recently become available, the low-MESF region relevant for EV analysis remains insufficiently covered by suitable reference particles. As a result, their use requires extrapolation of the calibrated fluorescence intensities into the EV range. Nevertheless, due to slight differences in the slope of the linear regression between different reference microparticle sets, caused by the intrinsic variation of assigning fluorescent values, extrapolation often results in substantial variations in the calculated MESF units of EVs^25^. Therefore, the utilization of submicron-sized fluorescent particles that better match the size and fluorescence properties of EVs can be gap-filling reference particles for FCM characterization of EVs. They would improve calibration accuracy and reduce the need for extrapolation.

Silica particles are often used as reference standards for various applications due to their ease of synthesis, excellent colloidal stability, and tunable particle sizes^26–29^. However, the fluorescent surface modification of submicron-sized silica particles, while maintaining colloidal stability is a challenging task that requires careful consideration of reaction conditions, such as solvent, reagent, reaction time, and temperature^30,31^. Furthermore, conventional multi-step approaches that involve solvent exchanges are time-consuming and often result in irreversible particle aggregation, highlighting the need for a simpler surface modification procedure.

A solution may be found in the application of silica-binding peptides (SBPs) or silica-forming peptides (SFPs), which are short amino acid sequences with high binding affinity for silica surfaces^32–35^. They offer simple and versatile functionalization of silica under mild aqueous conditions (pH 6–8) at room temperature. SBPs and SFPs bind to the silanol-rich surface of silica through various non-covalent interactions, including electrostatic effects, hydrogen bonding, and van der Waals forces^36–38^. The highly cationic 19-mer R5 peptide (SSKKSGSYSGSKGSKRRIL), derived from the silaffin-1 protein, is known for its ability to catalyze silica precipitation and is undoubtedly the most widely used and thoroughly investigated SFP^39–42^.

Reported here is the development of submicron-sized fluorescent silica particles as fluorescence reference materials for FCM characterization of EVs. The surface modification of the in-house synthesized silica particles was performed using FITC-labeled R5 peptides. After comprehensive characterization, the resulting particles were evaluated for their efficacy as fluorescent reference particles for the calibration of FCM measurements. Finally, calibration with these fluorescent silica particles was demonstrated using red blood cell–derived EVs labeled with FITC-conjugated anti-CD235 antibodies.

## Results and discussion

### Synthesis, characterization and fluorescent labeling of silica particles

**Fig. 1:**
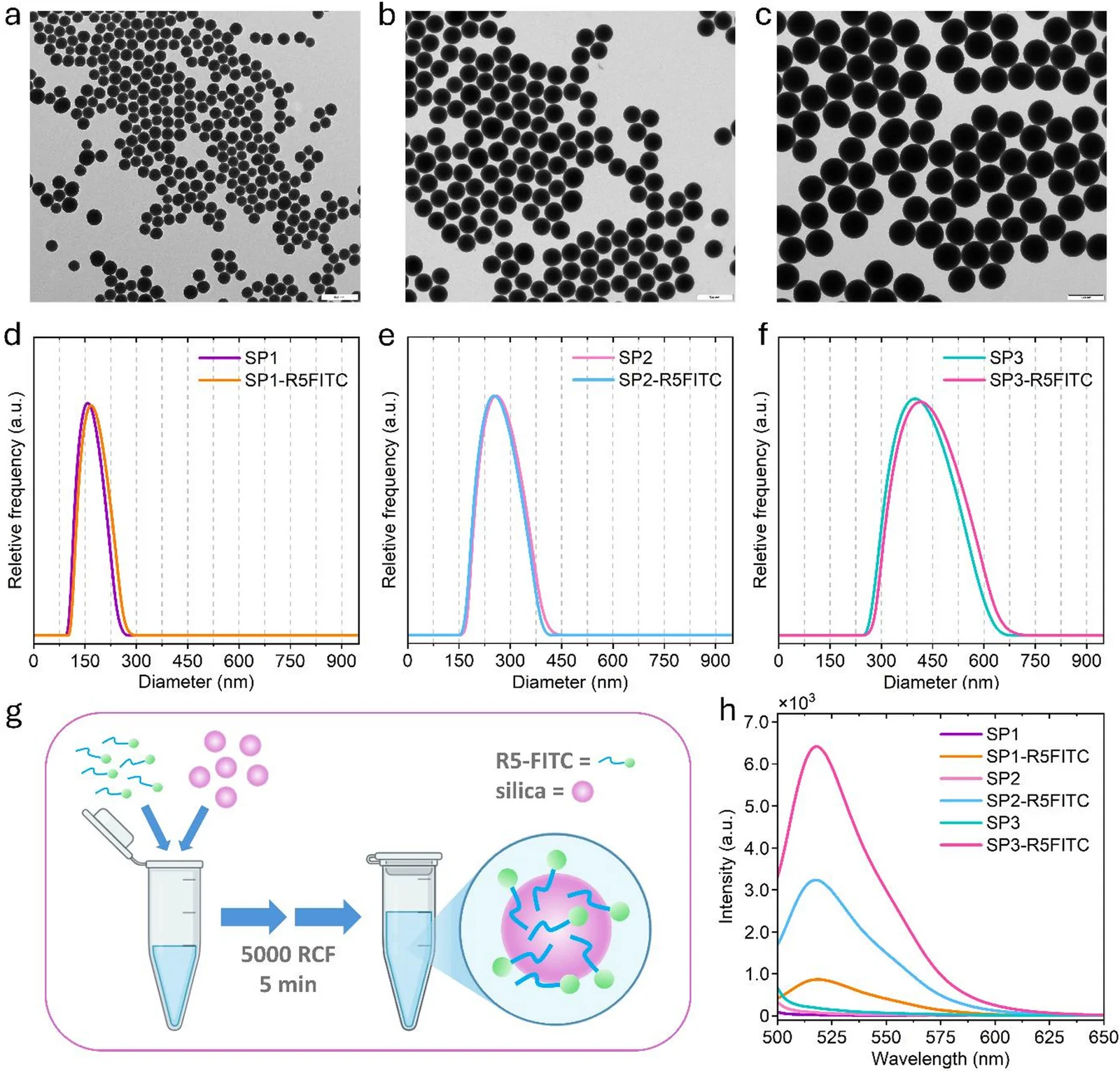
Characterization of blank and R5-FITC peptide-labeled silica particles. TEM images of (**a**) SP1, (**b**) SP2 and (**c**) SP3 silica particles (scale bars: 500 nm). DLS analysis of (**d**) SP1, (**e**) SP2 and (**f**) SP3 silica particles before and after fluorescent labeling. (**g**) Schematic illustration of fluorescent labeling with R5-FITC peptides. (**h**) Emission spectra of blank and R5-FITC peptide-labeled silica particles.

Submicron-sized silica particles of three different sizes (SP1 ~ 170 nm, SP2 ~ 260 nm, SP3 ~ 400 nm) were synthesized using the well-established Stöber method^43,44^. TEM images shown in Fig. 1a–c indicate that the resulting particles are well-dispersed and highly monodisperse. This was further confirmed by DLS analysis (Fig. 1d–f). Zeta potential measurements were used to assess the colloidal stability of the particles. The measured highly negative zeta potential values (Table 1) indicate excellent colloidal stability, which can be attributed to the dissociation of surface silanol groups, in good agreement with the literature^30,45^. For additional characterization of the blank silica particles (PTA, and AFM) see ref. 41, where traceable size and number concentrations were also determined via SAXS and spICP-MS, respectively (Table 1).

The silica particles were subsequently surface modified with R5-FITC peptides that were prepared under standard SPPS conditions in a continuous flow reactor (Fig. S1). The surface modification procedure is simple, fast and straightforward: an aqueous peptide solution (0.1 mg/mL) was added to the silica dispersion at room temperature, followed by purification via centrifugation (Fig. 1g). To remove the supernatant without disturbing the pellet, gel-loading pipette tips (VWRI732-3671, VWR) were used. Adjusting pH was not necessary. Based on DLS investigations, only insignificant changes in the mean hydrodynamic diameter of the silica particles were observed after functionalization, see Fig. 1d–f; Table 1. As anticipated, the surface modification resulted in a decrease in the zeta potential (Table 1) due to the binding of positively charged R5-FITC peptides to the highly negative silica surface, while at the same time preserving excellent colloidal stability. The emission spectra of the purified samples (Fig. 1h) confirmed successful surface modification, showing the expected emission peaks of FITC with a maximum at 518 nm. Particle concentration was kept constant for all samples during PL measurements. It is important to note that, during preliminary experiments, we successfully modified the surface of several commercially available silica particles with R5-FITC and R5-Cy5 peptides (Fig. S2). This demonstrates that R5 peptide is well-suited for modifying a wide range of nano- and submicron-sized silica particles with various fluorescent dyes.

**Table 1:** Overview of mean diameters determined by TEM and DLS, mean zeta potentials, and number concentrations.

Sample	TEM (nm) *	DLS (nm) **	Zeta potential (mV) **	spICP-MS (*10^15^ kg^− 1^)
SP1	174 ± 15	176 ± 31	-57.1 ± 0.4	3.34 ± 0.27
SP1-R5-FITC	–	187 ± 34	-52.4 ± 0.4	–
SP2	261 ± 14	293 ± 49	-67.5 ± 0.5	1.08 ± 0.16
SP2-R5-FITC	–	284 ± 49	-63.7 ± 0.3	–
SP3	404 ± 13	466 ± 72	-73.9 ± 0.2	0.74 ± 0.09
SP3-R5-FITC	–	459 ± 79	-71.1 ± 0.2	–

*N = 150.

**DLS and zeta potential measurements were conducted at pH 7.4.

### Isolation, fluorescent labeling and characterization of red blood cell-derived EVs (REVs)

**Fig. 2:**
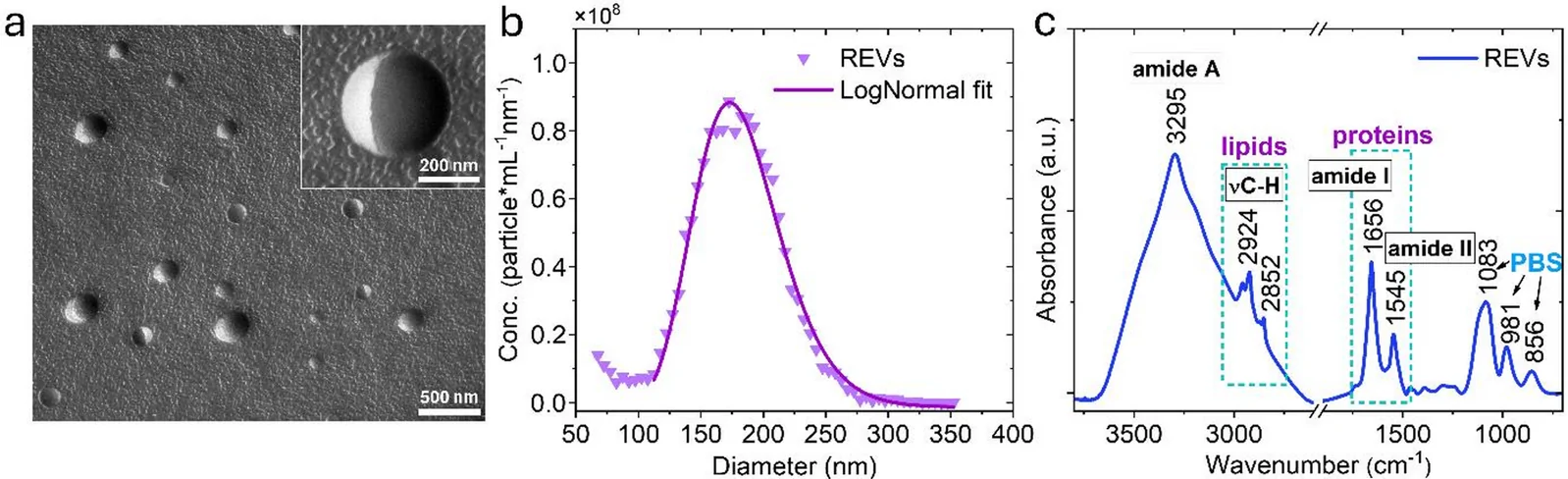
Characterization of REVs. (**a**) Representative FF-TEM image of REVs (scale bar: 500 nm). Inset: a single REV at higher magnification (scale bar: 200 nm). (**b**) Number-based size distribution of REVs determined by MRPS. (**c**) FTIR spectrum showing characteristic absorption bands of REVs.

REVs were isolated from whole blood according to our previously published protocol^46^. To visualize REVs in a hydrated environment, freeze-fracture transmission electron microscopy (FF-TEM) was applied. Figure 2a shows a typical FF-TEM image of a REV sample that reveals uniform particles with a spherical morphology; additional FF-TEM images are presented in Fig. S3. The average hydrodynamic diameter of REVs (223 ± 25 nm) was determined by DLS, see Fig. S4 in the Supplementary information. Counting and sizing of REVs were performed by microfluidic resistive pulse sensing (MRPS). MRPS is the nanoscale implementation of the Coulter principle in a microfluidic cartridge^47^. It is a non-optical, single-particle detection method. The particle size distribution was found to be approximately log-normal with a mean diameter of 179 nm with 36.3 nm standard deviation (Fig. 2b). The result is smaller compared to that obtained by DLS; however, MRPS does not measure hydrodynamic radius, unlike DLS and NTA. Instead, MRPS determines the volume of conductive ions displaced by each particle as it passes through a nanoconstriction in the fluid flow. The particle concentration, measured by MRPS, was 7.8 × 10^11^ 1/mL.

The FTIR spectrum (Fig. 2c) of the REV sample displays characteristic absorption bands corresponding to lipids and proteins. The bands at 2924 and 2852 cm⁻¹ are attributed to the C–H stretching vibrations of methylene groups in lipid acyl chains. Protein content is evidenced by the presence of the typical amide A, amide I, and amide II bands at 3295, 1656, and 1525 cm⁻¹, respectively, originating from the peptide backbone. Furthermore, the ratio of the area of the amide I band to that of the C–H stretching region (3020–2800 cm⁻¹) can serve as a quality control parameter for REV samples^48,49^. In our measurements, this ratio is 2.11 ± 0.03, typical for REV samples and consistent with previously reported values^50–52^.

Fluorescent labeling of EVs is commonly performed by targeting their transmembrane proteins using primary and secondary antibodies^53^. In this study, REVs were labeled by FITC-conjugated monoclonal antibodies (anti-CD235a-FITC).

### FCM investigations

**Fig. 3:**
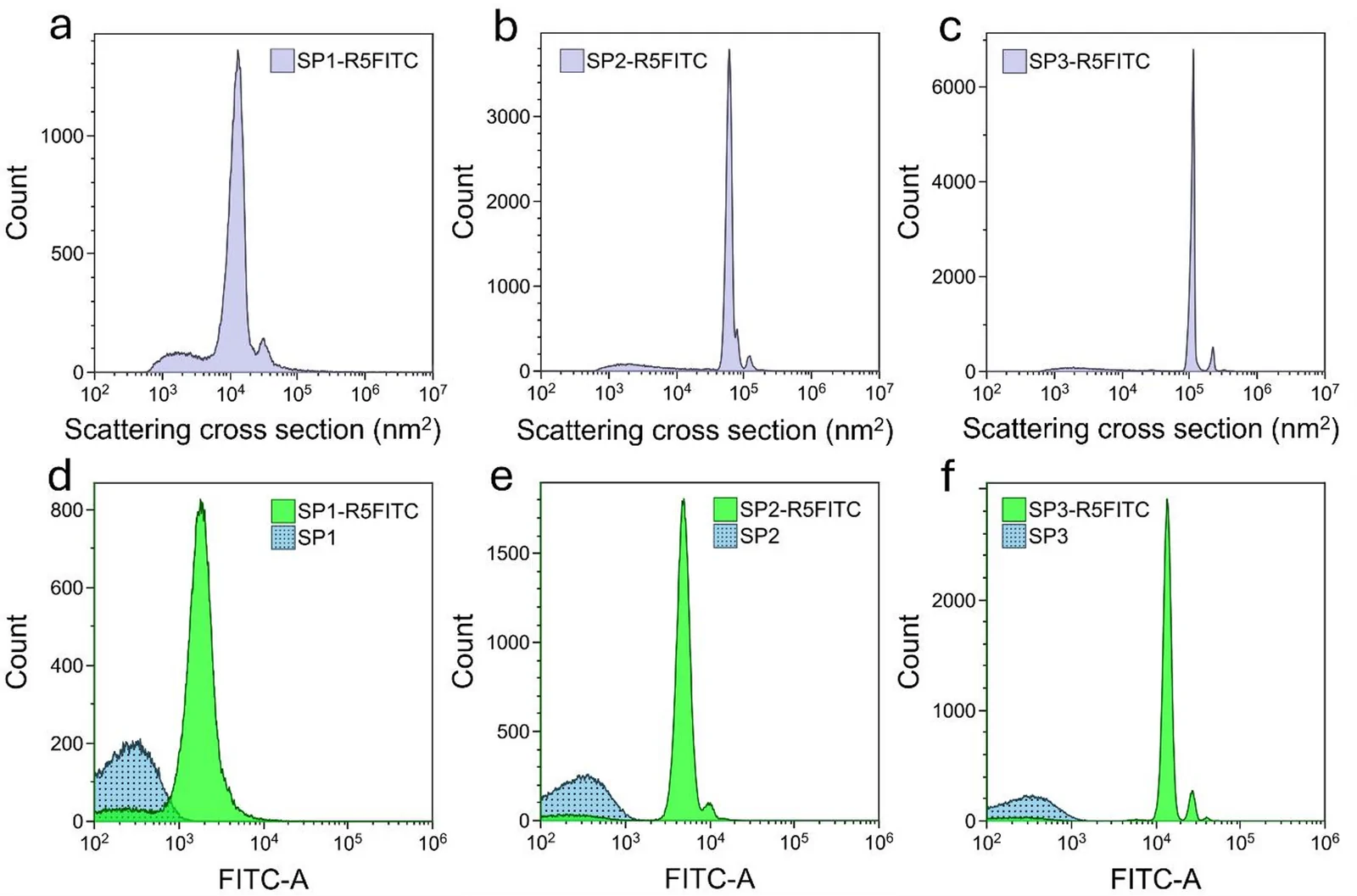
FCM analysis of silica particles. (**a**–**c**) Scattering cross-section distribution profiles of R5-FITC peptide labeled silica particles. (**d**–**f**) Overlays of FITC-A histograms of blank and R5-FITC peptide-labeled silica particles.

Once characterized, the silica particles were investigated using FCM. As discussed above, the fluorescence intensity in arbitrary units depends on various experimental parameters, such as the optical configuration and the gain factor of the detector. Therefore, the calibration from arbitrary units to absolute units of MESF is necessary. Typical FCM histograms display a single measurement parameter (e.g., side scatter intensity (SSC-A), forward scatter intensity (FSC-A), fluorescence intensity (FITC-A)) on the x-axis and the number of events on the y-axis. Figure 3a–c show the scattering cross section profile of the R5-FITC-labeled silica samples in the violet SSC channel indicating a monodisperse particle population. Side-scatter intensities were calibrated to scattering cross-section values using the traceably characterized blank silica particles and Mie theory implemented in the FCMPASS software^54^. The histograms of the labeled silica particles in the FITC channel (Fig. 3d–f) show a clear shift toward higher intensities, indicating successful fluorescent labeling. Importantly, the narrow peaks suggest homogeneous fluorophore distribution. As a negative control, Fig. 3d–f depict the background fluorescence of the blank silica particles in the same channel, which was subtracted from the fluorescence of the R5-FITC-labeled silica samples. It is also evident that as particle size increases, the measured median fluorescence intensity (MFI) increases, likely because the larger surface area allows more R5-FITC peptide to bind. The MESF values of the R5-FITC-labeled silica particles were determined by comparing their fluorescence intensity signal (Fig. 1h) to that of a solution of fluorescein of known concentration (Fig. S5), normalized by the particle concentration determined previously via spICP-MS (Table 1)^44^. Emission spectra were corrected for the background (~ 3%) from blank particles. It is important to note that fluorescein and its derivatives exhibit highly pH-sensitive absorption and emission^55^, therefore fluorescent calibration and FCM measurements were performed in the same buffer (10 mM HEPES) at pH 7.4^56^. The measured MFI values and the corresponding MESF values of the labeled particles are summarized in Table 2. It should be noted that these values are indicative only. Although the particle number concentration was previously determined traceably by SAXS and spICP-MS, a complete uncertainty budget for the MESF values cannot currently be established because the fluorescence intensity of the particles has not been characterized traceably. Traceable determination of the absolute fluorescence quantum yield would enable rigorous estimation of the uncertainty associated with the MESF values; however, such measurements are beyond the scope of the present study and will be performed in future work.

**Table 2:** MFI and MESF values of R5-FITC peptide labeled silica particles.

Sample	MFI	MESF
SP1-R5-FITC	1520	278
SP2-R5-FITC	4564	1046
SP3-R5-FITC	13,279	2053

**Fig. 4:**
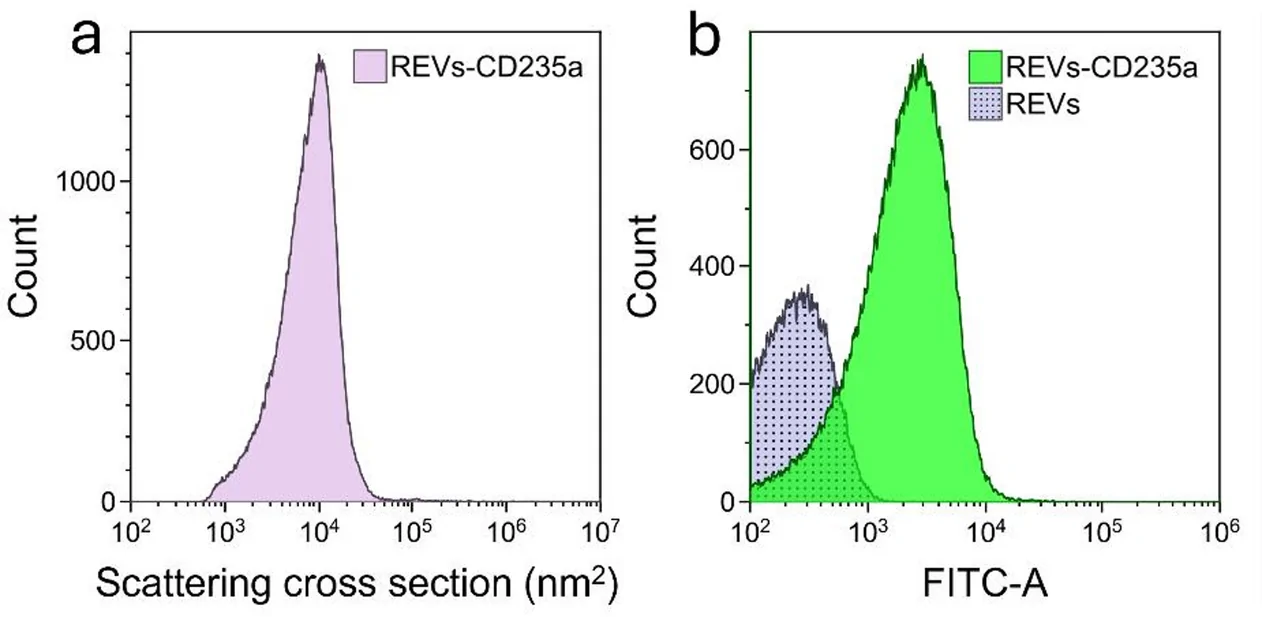
FCM analysis of REVs. (**a**) Scattering cross-section distribution profile of anti-CD235a-FITC-labeled REVs. (**b**) Overlays of FITC-A histograms of non-labeled and anti-CD235a-FITC-labeled REVs.

After determining the MESF values of the R5-FITC-labeled silica particles, we analyzed the FITC-labeled REV samples in the violet SSC channel (Fig. 4a). which revealed a scattering intensity similar to that of fluorescent silica particles. It also suggests a size distribution broader than that of the silica particles. To enable quantitative comparison, the side-scatter intensities were calibrated to units of scattering cross section as described above. Notably, the relatively high scattering intensity of REVs compared to similarly sized EVs may arise from their hemoglobin-rich lumen, which likely increases the effective RI. In contrast to silica particles, REVs may exhibit a complex refractive index due to the presence of hemoglobin, resulting in optical properties that differ from those of homogeneous dielectric particles. Consequently, although the calibrated scattering cross-section values allow instrument-independent comparison of optical signals, conversion of REV scattering signals to particle diameter was not performed because the optical properties of REVs are insufficiently characterized for accurate sizing. Instead, REV size distribution and particle concentration were determined independently by MRPS measurements (Fig. 2b).

**Fig. 5:**
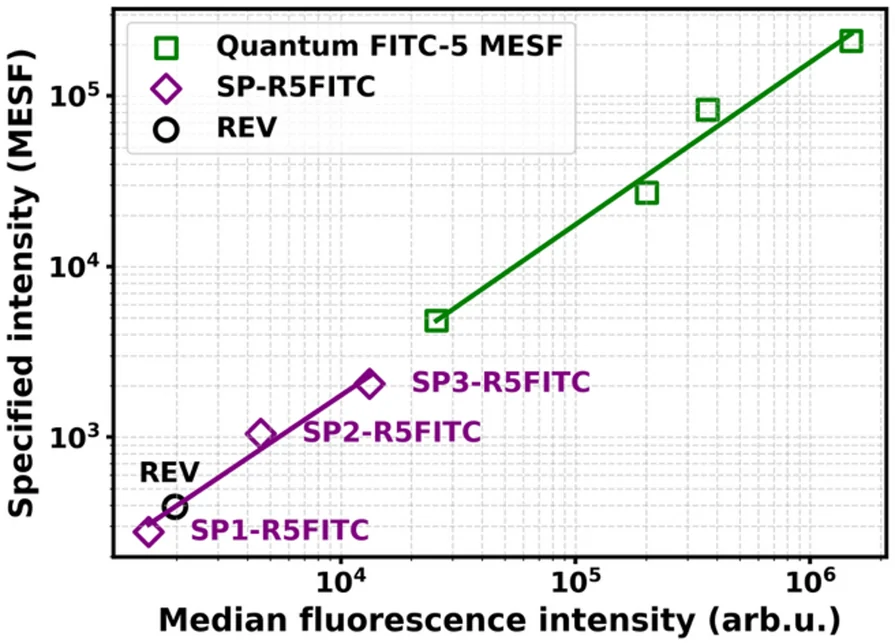
Correlation between MFI and MESF. MESF calibration of anti-CD235a-FITC antibody-labeled REVs using R5-FITC-labeled silica particles and fluorescence calibration curve of commercially available calibration microparticles.

The histogram of the labeled REV sample in the FITC channel (Fig. 4b) shows a clear shift towards higher intensity values, indicating successful labeling of the REVs with anti-CD235a-FITC antibodies. As a negative control, Fig. 4b shows the background fluorescence of the non-labeled REVs in the same channel (see Fig. S6 for additional FCM control data). The background-corrected MFI value of the labeled REVs was 1967 a.u. Next, a fluorescence calibration curve from MFI to absolute units of MESF was prepared by plotting the fluorescent silica particles’ MESF units against their MFI values, and the MESF value of the anti-CD235a-FITC-labeled REVs was determined (Fig. 5).

Figure 5 also shows the fluorescence calibration curve of commercially available calibration microparticles (Quantum™ FITC-5 MESF). The comparison shows that commercial beads are not suitable for calibration in the low MESF (less than 10,000) range without extrapolation, where the MFI of the antibody-labeled REVs is observed. However, using the calibration based on the R5-FITC-labeled silica particles, the assignment of MESF values to the measured fluorescence intensity of REVs is feasible. The anti-CD235a-FITC antibody labelled REVs’ fluorescence intensity was 390 MESF. It should be noted that MESF values can be used to estimate antigen copy numbers; however, fluorophore conjugation may alter the antibody’s effective fluorescence yield, introducing systematic uncertainty. More accurate estimation of antigen copy number could be achieved using antibody-capture beads with known antibody-binding capacity (ABC) values, but this approach was beyond the scope of the present study.

The investigated REV model system may not fully represent the optical properties of average EV populations. In addition, REVs are expected to express relatively high levels of CD235a, potentially resulting in greater antibody binding than is observed for many other EV-associated antigens. Nevertheless, the labeling strategy presented here could readily be adapted to produce particles with substantially lower peptide-to-particle ratios, consequently lower MESF values. In the present work, the achievable fluorescence brightness was primarily limited by the detection sensitivity of the flow cytometer used. The main goal of this study was to demonstrate a novel fluorescent particle labeling approach that may serve as the basis for future EV-like fluorescence reference materials, although traceable characterization of the fluorescent properties would still be required for metrological applications. Furthermore, this peptide-based fluorescent labeling strategy could be extended to other silica-based EV mimetics, including beads, which possess refractive index distributions closer to those of typical EVs, thereby enabling the simultaneous calibration of scattering and fluorescence signals using a single reference material^57,58^.

## Conclusions

Demonstrated here is the development and application of submicron-sized fluorescent silica particles as fluorescence reference materials for FCM characterization of EVs. The in-house synthesized silica particles were labeled with R5-FITC peptides in a simple, fast and straightforward manner. Importantly, the resulted particles exhibited homogeneous fluorophore coverage while preserving their excellent colloidal stability and high monodispersity. After comprehensive characterization, FCM analysis was conducted and the MESF values of the particles were determined. Most importantly, the R5-FITC peptide-labeled silica particles were suitable for the MESF calibration of the fluorescence of antibody-labeled EVs without the need for extrapolation. Therefore, the utilization of submicron-sized fluorescent silica particles that better match the size and fluorescence properties of EVs can be gap-filling reference particles in the field of EV characterization by FCM.

## Materials and methods

### Chemicals

1,8-Diazabicyclo[5.4.0]undec-7-ene (DBU, ≥ 98%), piperidine (≥ 99%), trifluoroacetic acid (TFA, ≥ 99%), 1-[bis(dimethylamino)methylene]-1 H-1,2,3-triazolo[4,5-b]pyridinium 3-oxide hexafluorophosphate (HATU, ≥ 98%), DL-dithiothreitol (DTT, ≥ 99.0%), N, N-diisopropylethylamine (DIPEA, ≥ 99%), and fluorescein (European Pharmacopoeia reference standard, 99.2%) were purchased from Merck. TentaGel^®^ R RAM resign (0.19 mmol/g) was obtained from Rapp Polymere. L-arginine (reagent grade, ≥ 98%), triisopropylsilane (TIS, 98%), and tetraethyl orthosilicate (TEOS, reagent grade, 98%) were purchased from Sigma-Aldrich. Ethanol absolute (EtOH, HiPerSolv CHROMANORM^®^, ≥ 99.8%) and ammonia solution (AnalaR NORMAPUR^®^, 25%) were obtained from VWR. Cyclohexane (cHex, 99.99%) was purchased from Lach-Ner. 200-nm-sized silica particles (AN200, 50 mg/mL) were obtained from Alpha Nanotech. 800-nm-sized silica particles (K800, 50 mg/mL) were obtained from Kisker Biotech. Experiments were conducted using high-purity deionized water (18.2 MΩ·cm). All reagents were used as received.

### Peptide synthesis

Peptide synthesis was carried out using standard solid-phase peptide synthesis (SPPS) conditions based on Fmoc chemistry. The peptide chains were elongated on TentaGel R RAM resin^59^ with a Rink amide linker on a 0.4 mmol scale manually. Coupling was performed in two steps. In the first step, three equivalents of Fmoc-protected amino acid, three equivalents of HATU^60^, and six equivalents of DIPEA were mixed in DMF and the mixture was shaken for 3 h. The second coupling was performed with one equivalent of amino acid, one equivalent of HATU, and two equivalents of DIPEA. After the coupling steps, the resin was washed three times with DMF, once with methanol, and three times with DCM. No truncated sequences appeared under these coupling conditions. The deprotection was performed in two steps with 15- and 5-minute reaction times with 2% DBU and 2% piperidine in DMF.

FITC was coupled after completion of the sequence. Thereafter, the cleavage was carried out with TFA/water/dl-dithiothreitol (DTT)/TIS at 0 °C for 1 h. The purification was performed by reverse-phase semi-preparative HPLC, using a Phenomenex Luna C18 100 Å 10 μm column (10 mm × 250 mm), the solvent system used was as follows: 0.1% TFA in water; 0.1% TFA, 80% acetonitrile in water; linear gradient was used during 60 min, at a flow rate of 4.0 mL min^− 1^, with detection at 206 nm. The fractions’ purities were determined using analytical HPLC with a Phenomenex Luna C18 100 Å 5 μm column (4.6 mm x 250 mm), and the pure fractions were pooled and lyophilized, followed by characterization using HPLC-MS.

### Synthesis of silica particles

The synthesis and characterization of silica particles are reported elsewhere in detail^44^. Briefly, ammonia solution, water, and ethanol were added into a 500 mL borosilicate glass bottle at ratios indicated in Table 3. Next, TEOS was added, and the solution was stirred at 350 RPM overnight under ambient conditions. The resulting silica particles were centrifuged (see details in Table 1) and washed three times with EtOH. Finally, the particles were redispersed in water.

**Table 3:** List of synthesis parameters.

Sample	EtOH (mL)	NH_3_ (mL)	DI (mL)	TEOS (mL)	RCF (g)
SP1	250	12.7	4.23	6.2	10,000*
SP2	250	25.3	11.25	7.5	7000*
SP3	250	50.6	16.9	25.3	5000*

*10 min, RT.

### Fluorescent labeling of silica particles

In a typical procedure, 40 µL of R5-FITC aqueous solution (0.1 mg/mL) was added to 25–40 µL of aqueous silica dispersion, mixed thoroughly, and incubated at room temperature in the dark for 2 min. Subsequently, the sample was centrifuged (2 × 5 min at 5000 RCF) and was washed with water. Finally, the pellet was redispersed in 1 mL of 10 mM HEPES (pH 7.4) and stored in the dark.

#### Ethical approval and consent to participate

The Medical Research Council of Hungary (ETT TUKEB IV/701-3/2022/EKU) provided ethical approval for the use of human blood samples, and all procedures were carried out in accordance with the Declaration of Helsinki. Written informed consent was obtained from the volunteer blood donor.

### Isolation of red blood cell-derived EVs (REVs)

REVs were isolated according to our standard protocol^46^, which is consistent with the recommendations of the International Society of Extracellular Vesicles outlined in the MISEV2023 guidelines^61^. Briefly, 12 mL of a blood sample was collected from a healthy donor in K_3_EDTA blood collection tubes (Greiner Bio-One) and centrifuged (800 RCF for 10 min at 4 °C). Red blood cells (RBCs) were collected, and the supernatant (platelet-rich plasma) was discarded. Next, RBCs were washed three times with 5 mL of physiological saline solution and centrifuged (800 RCF for 10 min at 4 °C) to remove residual platelets and buffy coat. Then, 5 mL of PBS (pH 7.4) was added, and RBCs were incubated at 4 °C for a week to produce EVs. This was followed by two consecutive sedimentations (2500 RCF for 10 min and 3000 RCF for 15 min at 4 °C, respectively). Next, the aliquoted supernatant was pelleted (16,000 RCF for 30 min at 4 °C), and the pellets were resuspended in 0.5 mL of PBS. A final purification step was performed using an Izon qEVoriginal (70 nm) SEC column, following the manufacturer’s protocol. After loading the EV sample, elution was performed using 10 mM PBS solution (pH 7.4). The void volume was set to 2.7 mL, and 0.5 mL fractions were collected. Fraction 2, containing the highest concentration of REVs, was kept at 4 °C until further investigations.

### Fluorescent labeling of REVs

6 µL of SEC-purified REV sample and 86 µL of PBS were mixed thoroughly in a 0.5 mL amber microcentrifuge tube. Next, 8 µL of anti-CD235a-FITC (Invitrogen 11-9987-82, eBioscience™, 0.5 mg/mL) antibody solution was added. To validate specificity, a matched isotype control mixture was prepared: 6 µL of SEC-purified REV sample and 74 µL of PBS were mixed, and 20 µL of FITC isotype control (Invitrogen 11-4732-42, eBioscience™, 0.2 mg/ml) was added. Samples were incubated at room temperature for 60 min on an orbital shaker.

**Transmission electron microscopy (TEM)** images were obtained using a FEI Morgagni 268D electron microscope operating at 80 kV. Diluted silica samples were dropped and dried on 200 mesh formvar coated copper grids.

**Freeze-fracture transmission electron microscopy (FF-TEM)** allows the morphological study of REVs in an aqueous medium. To avoid freezing artifacts, REV samples were mixed with glycerol at a 3:1 volume ratio. 2 µL sample was pipetted onto a gold sample holder, subsequently frozen in liquid freon, and then stored in liquid nitrogen. Fracturing was carried out at -100 °C using a Balzers BAF 400D freeze-fracture device. Carbon-platinum shadowing was used to make replicas from the fractured surfaces, followed by washing with surfactant solution and water. Replicas were placed on 200 mesh copper grids and analyzed by TEM.

**Dynamic light scattering (DLS)** measurements were conducted at 20 °C using an Anton Paar Litesizer 700 instrument equipped with a 658 nm laser diode. Measurements were performed in polystyrene Sarstedt cuvettes (67.745), and the data were evaluated using the Kalliope software. Samples were diluted in 10 mM HEPES buffer (pH 7.4).

**Microfluidic resistive pulse sensing (MRPS)** measurements were conducted by a Spectradyne nCS1 instrument. Measurements were conducted using factory-calibrated C400 cartridges with a measurement range of 65 nm to 400 nm. Samples were diluted 100-fold with PBS containing 0.05 w/v% Poloxamer 188,pre-filtered using 100 kDa MWCO Amicon Ultra 0.5 mL filters (Merck Millipore, Germany).

**Fourier-transform infrared spectroscopy (FTIR)** spectra were recorded using a Varian 2000 FT-IR Scimitar Series spectrophotometer equipped with a Golden Gate single-reflection ATR unit. 3 µL REV sample was pipetted onto the ATR crystal, and a thin film was formed by gently evaporating the solvent under a stream of N₂. Typically, 64 scans were acquired at a nominal resolution of 2 cm⁻¹.

**Zeta potential** measurements were performed at 20 °C using an Anton Paar Litesizer 700 instrument equipped with a 658 nm laser diode. Measurements were conducted using polycarbonate Anton Paar Omega cuvettes (Mat. No. 225288), and data were analyzed with the Kalliope software. Samples were diluted with 10 mM HEPES buffer (pH 7.4) before measurements.

**Ultraviolet–visible spectroscopy (UV-Vis)** was used to determine the concentration of the fluorescein stock solution estimated by mass. Measurements were performed at room temperature using a Hewlett-Packard 8453 diode array spectrophotometer with standard 10 mm optical path quartz glass cuvette in 100 mM sodium borate buffer (pH 9.5). The concentration was determined using the Beer–Lambert law at 490 nm with a molar absorption coefficient of 8.70 × 10^4^ M⁻¹·cm⁻¹^62^.

**Fluorescence emission** spectra were measured at 20 °C using a Jasco FP-8500 fluorimeter using 488 nm excitation. Fluorescein and silica samples were diluted in 10 mM HEPES buffer (pH 7.4). Particle concentration was set to 9.25 × 10⁹ particles per 2 mL. MESF units were determined by comparing the fluorescence intensity signal from the fluorescently labeled silica particles to the signal from fluorescein solutions.

**Flow cytometry (FCM)** experiments were conducted on a Beckman Coulter CytoFLEX S V4-B2-Y0-R3 flow cytometer, equipped with 405 nm, 488 nm, and 638 nm lasers. For the measurement of single particles, Violet-Side Scatter (VSSC) detection was used. This VSSC channel served as the trigger, with a threshold of 3000 arbitrary units. The detector gain was set to 250 for the VSSC channel and to 2000 for the FITC and 1000 for the APC fluorescence channels. Each sample was analyzed for 60 s at a flow rate of 10 µL/min. EV samples were diluted 3.6 × 10^3^-fold in 10 mM HEPES–150 mM NaCl buffer (pH 7.4) prior to measurements. Silica samples were diluted 4.0 × 10^4^ – 3.6 × 10^5^-fold in 10 mM HEPES buffer (pH 7.4) prior to measurements. Data were evaluated using the Kaluza software. For additional experimental details, see the MIFlowCyt checklist and a detailed MIFlowCyt-EV framework in the Supplementary information.

## Supplementary Information

Below is the link to the electronic supplementary material.


Supplementary Material 1


## Data Availability

The datasets used and/or analyzed during the current study are available from the corresponding authors on reasonable request.
