# Supplementary material for: Low-MESF fluorescent reference particles for flow cytometry analysis of extracellular vesicles
Source: Sci Rep. 2026 Sep 1;16:27406. doi: 10.1038/s41598-026-60804-3 (PMC13534728; doi:10.1038/s41598-026-60804-3)
Supplement: Supplementary file 1 [file 41598_2026_60804_MOESM1_ESM.pdf]

Supplementary information for

Low-MESF fluorescent reference particles for flow cytometry analysis of extracellular vesicles

Marcell Pálmai[1], Viet Thuc Dang[2], Anikó Gaál[1], Kinga Ilyés[1,3] István Mándity[1], Judith Mihály[1], Andy I. Nguyen[2], Preston T. Snee[2,], Zoltán Varga[1,4,*]*

[1]Institute of Materials and Environmental Chemistry, HUN-REN Research Centre for Natural Sciences, Budapest, Hungary

[2]Department of Chemistry, University of Illinois Chicago, Chicago, IL, United States

[3]Hevesy György PhD School of Chemistry, Eötvös Loránd University, Budapest, Hungary

[4]Department of Physical Chemistry and Materials Science, Budapest University of Technology and Economics, Budapest, Hungary

*Correspondence: Preston T. Snee (sneep@uic.edu)

Zoltán Varga (varga.zoltan@ttk.hu)

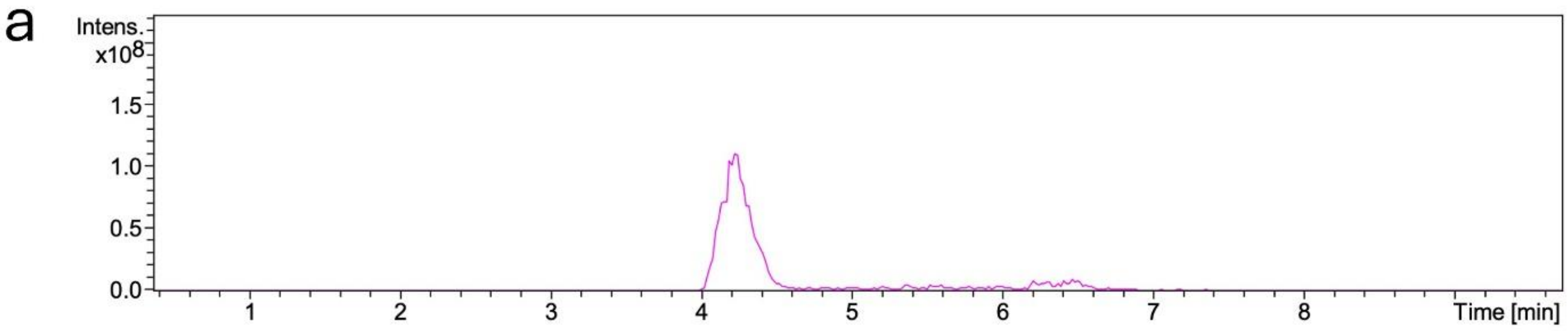

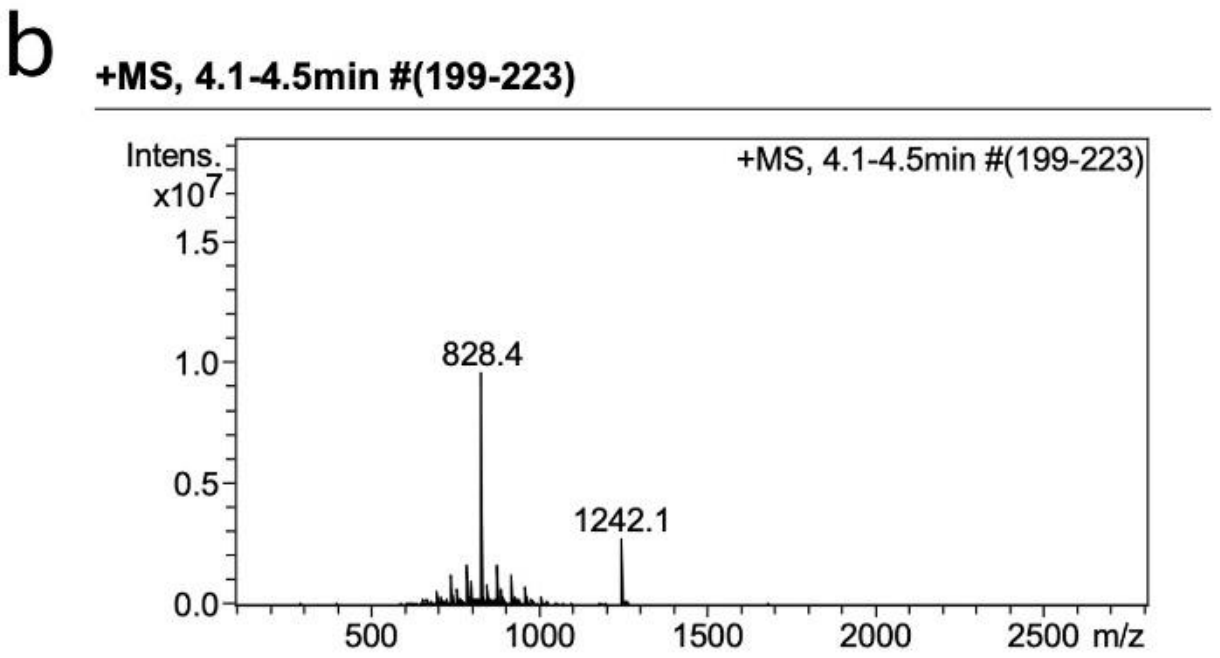

c

#	m/z	I
1	740.2	1261492
2	784.2	1703909
3	828.4	9574848
4	829.0	3360853
5	829.3	1593824
6	872.2	1643602
7	916.2	1218553
8	1241.6	1910228
9	1242.1	2705936
10	1242.6	1947586

Fig. S1. HPLC-MS analysis of R5-FITC peptide. (a) Representative chromatogram, (b) mass spectrum, and (c) mass list of R5-FITC peptide.

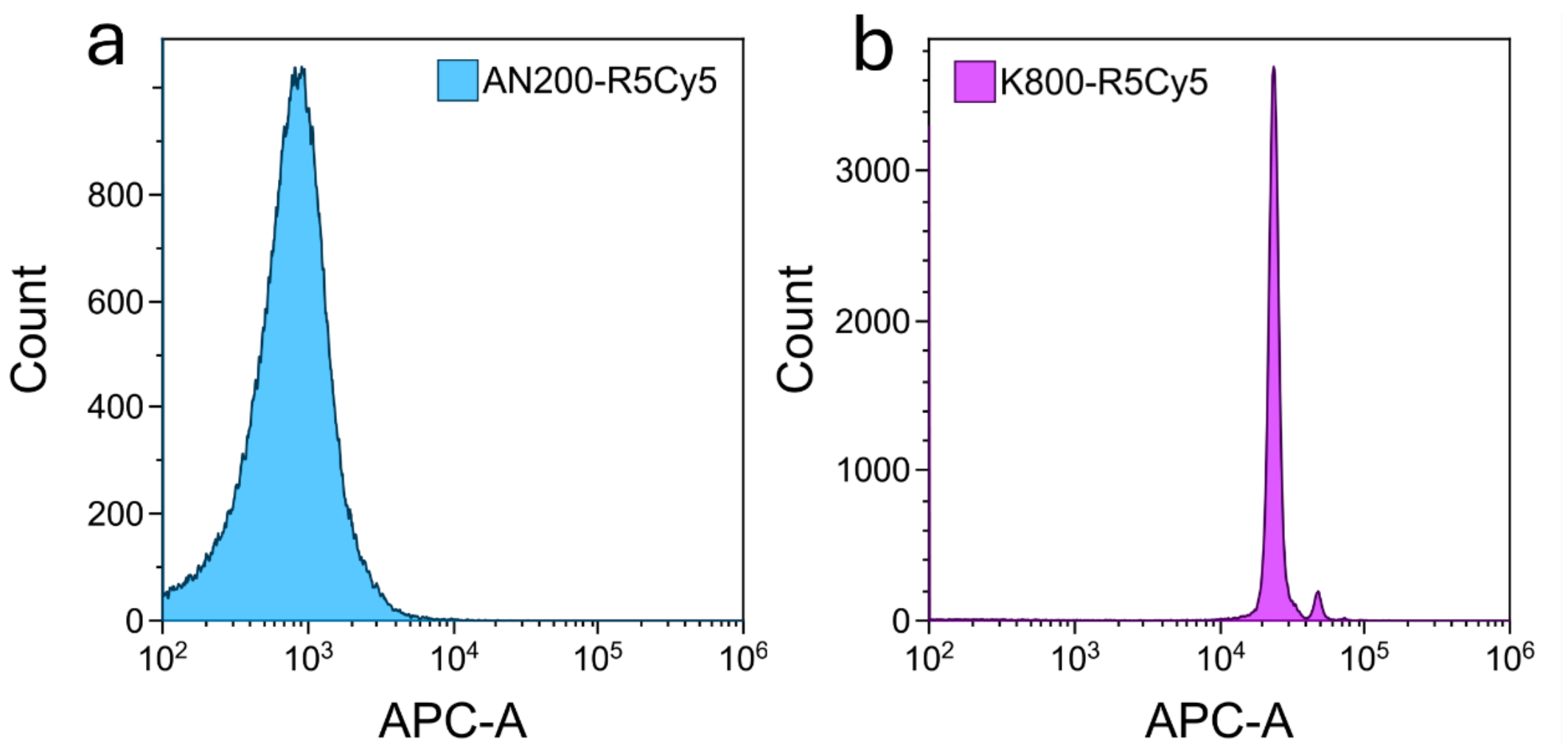

Fig. S2. FCM analysis of commercial silica particles. R5-Cy5 peptide-labeled (a) 200- and (b) 800-nm-sized silica particles in the APC channel (excitation: 638 nm).

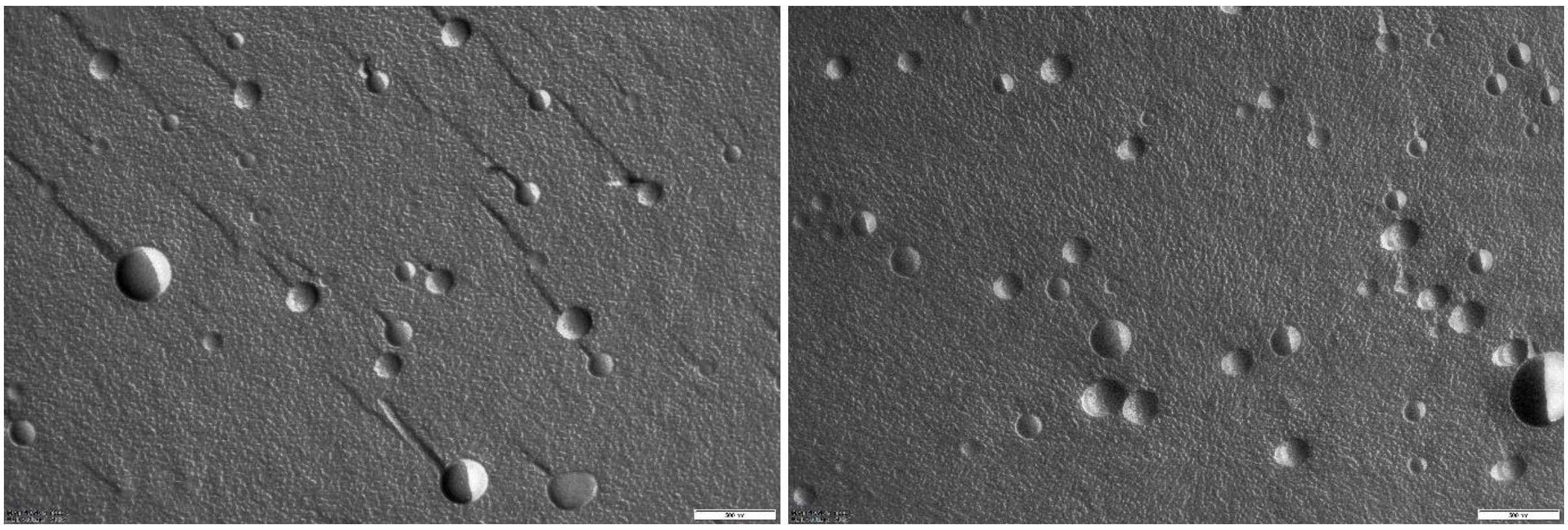

Fig. S3. Additional FF-TEM images of REVs (scale bars: 500 nm).

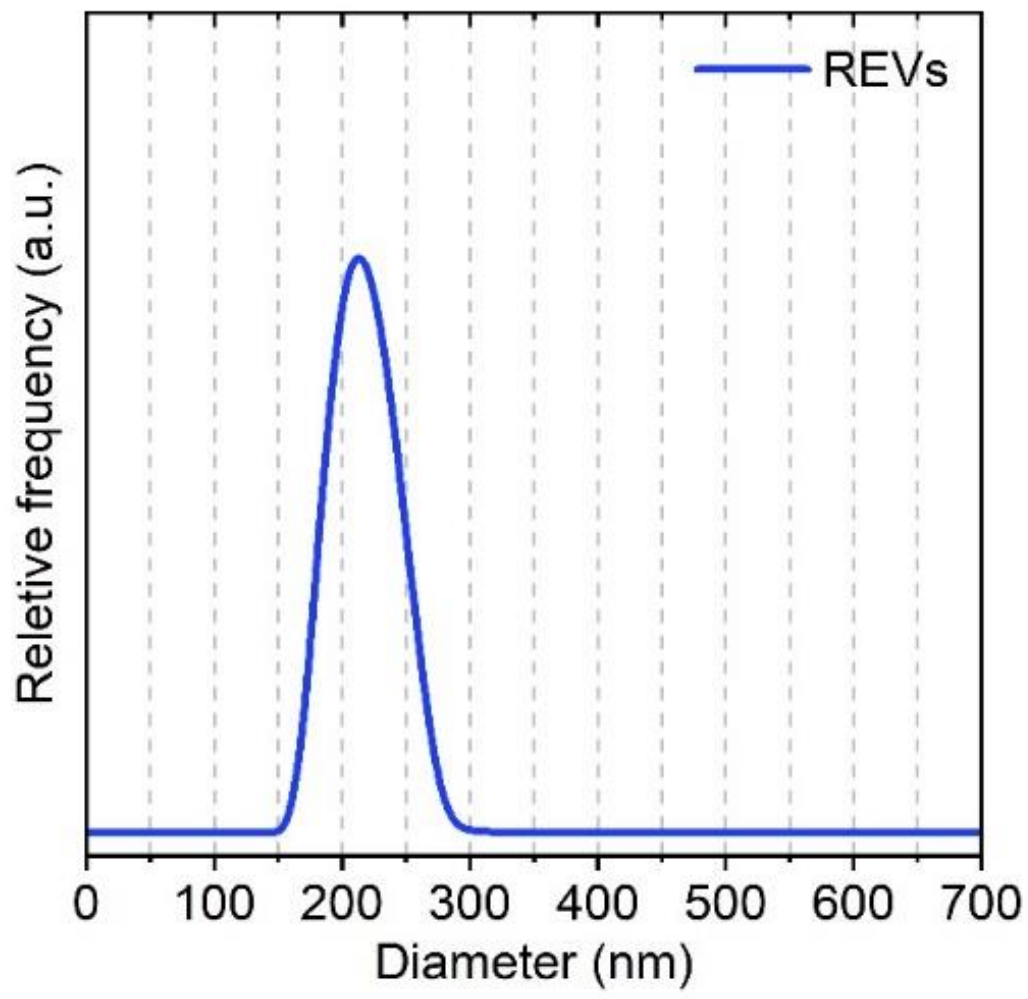

Fig. S4. DLS analysis of REVs.

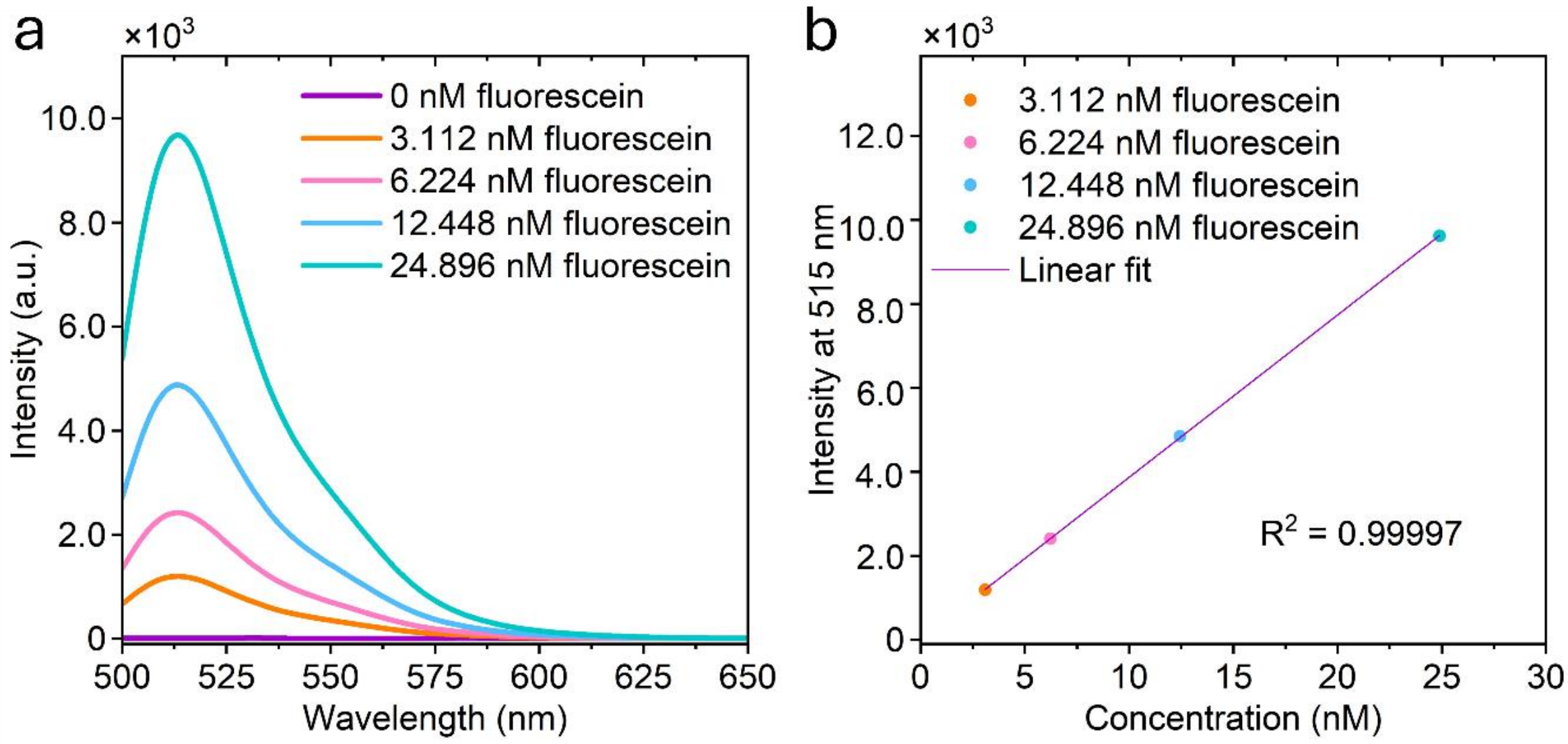

Fig. S5. (a) Emission spectra of fluorescein solutions (10 mM HEPES buffer, pH 7.4) and the corresponding (b) fluorescent intensity calibration curve.

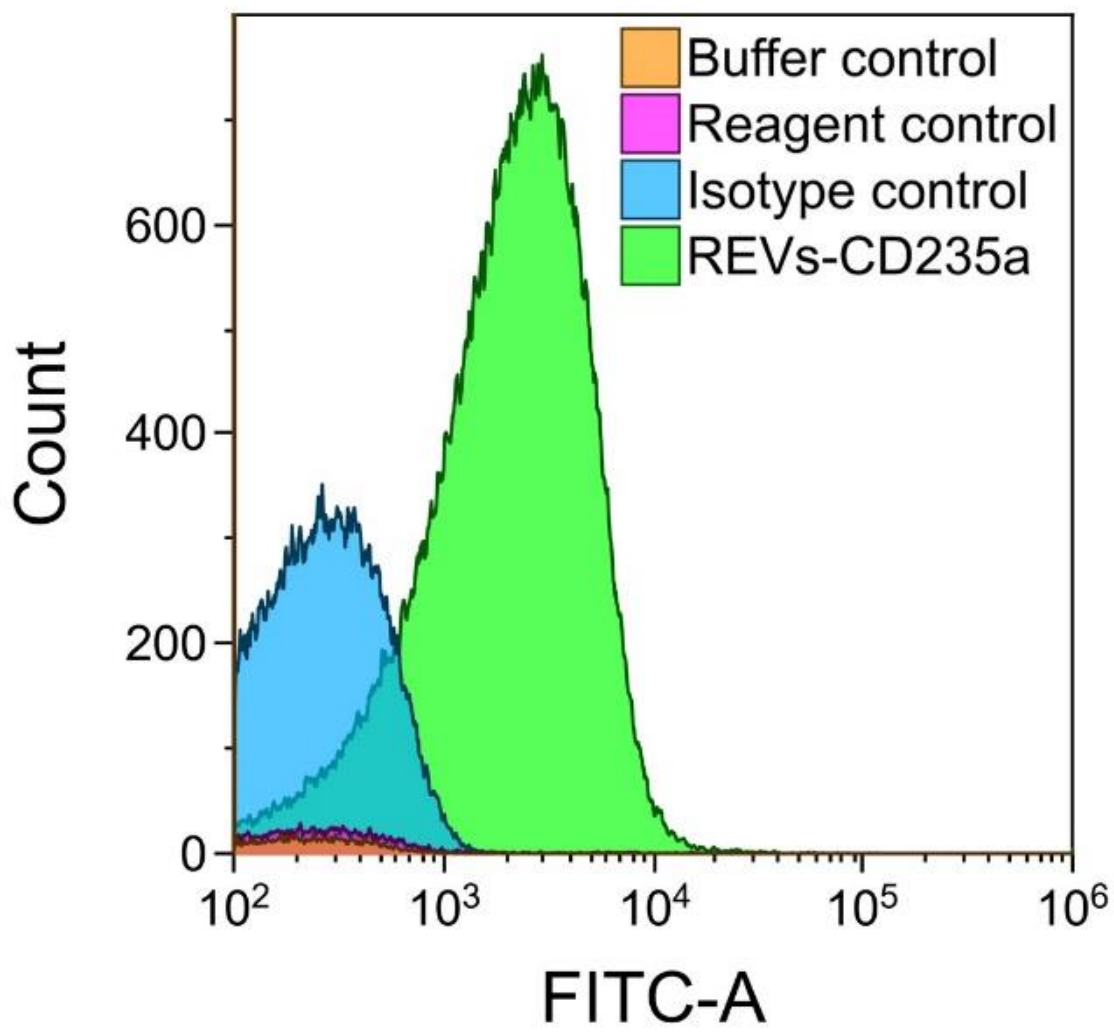

Fig. S6. Overlays of FITC-A histograms of buffer control, reagent control, isotype control, and anti-CD235a-FITC-labeled REVs.

R5-Cy5 peptide synthesis

Peptides were synthesized on a 0.1 mmol scale using the CEM Liberty Blue automated peptide synthesizer. The solvent for all SPPS solutions was DMF unless otherwise stated. The prepared amino acid, Oxyma, and DIC concentrations were 0.20 M, 1.0 M, and 1.0 M respectively. The deprotection solution was 20% (v/v) 4-methylpiperidine in DMF.

1. **Swelling:** Rink MBHA amide resin was allowed to swell in 5 mL DMF at 90° C while being agitated with N_2 gas for 5 min, and then it was drained.
2. **Single coupling 0.1 mmol scale:** A 2 mL volume of 4-methyl-piperidine in DMF (20% v/v) was added to the swelled resin and agitated with N_2 gas for 15 s at 90° C, and then it was drained. The resin was washed 4 times, each by adding 5 mL DMF, agitating with N_2 for 5 s, and then drained. 1.0 mL of 1.0 M DIC and 0.5 mL of 1.0 M Oxyma was added to the reaction vessel containing the resin followed by 2.5 mL of 0.20 M amino acid solution. After 15 s to warm up to 75° C followed by 110 s held at 90° C with N_2 gas agitation, the reaction vessel was drained. The resin was washed 4 times, each by adding 5 mL DMF, agitating with N_2 for 5 s, and drained.
3. **Final deprotection:** The N-terminal amino acid was deprotected with 4 mL of 20% (v/v) 4-methyl-piperidine for 50 s then drained. The resin was then washed 4 times with 4 mL of DMF and drained.

Cleavage from solid support and side chain deprotection

The resin was transferred to a 5 mL fritted polypropylene cartridge and then washed with 5 mL DCM while being swirled by hand for 30 s and then drained. The DCM wash was done twice more, after which the resin was air dried. The resin was transferred to a scintillation vial, and a 10 mL cleavage cocktail of 95% TFA, 2.5% TIPS, and 2.5% water was added to the resin. The mixture was shaken for 3 hr, filtered through a fritted polypropylene cartridge, and the resin was washed with TFA (3 times 1 mL) followed by DCM washes (3 times 1 mL). The filtrate and washings were collected into a round bottom flask and dried under vacuum to yield an oily residue.

Ether precipitation

Diethyl ether (~20 mL) was added to the residue and the mixture was sonicated to obtain a white precipitate, which was transferred to a centrifuge tube and separated from the liquid phase by centrifugation for 3.0 min at 3.0 RCF, forming a white pellet at the bottom of the centrifuge tube. The liquid phase was then poured off and the pellet was washed twice more with ~20 mL of diethyl ether, each time breaking up the pellet by sonication, and the crude product was allowed to air dry.

Reverse phase high performance liquid chromatography (RP-HPLC)

Preparative RP-HPLC was performed on a Waters Prep 2545 HPLC, equipped with a C18 (X-Bridge Peptide BEH 5μm, 19x 100 mm) column at a 17 mL/min flow rate monitored at a 214 nm wavelength. Crude samples were dissolved in water and filtered through a 0.2 μm filter before RP-HPLC purification. RP-HPLC was performed with water as the weak solvent and acetonitrile as the strong solvent. HPLC solvents were acidified with 0.1% trifluoracetic acid. Fractions were analyzed for the product using UPLC-MS. Fractions containing the product were dried by lyophilization.

MIFlowCyt checklist

Requirement	Please Include Requested Information
1.1. Purpose	The purpose of the study was to develop submicron-sized fluorescent silica particles as fluorescence reference materials for the calibration of FCM measurements in EV analysis. Silica particles functionalized with FITC-labeled R5 peptides can provide fluorescence properties more comparable to labeled EVs than conventional reference microparticles, thereby improving calibration accuracy and reducing the need for extrapolation in EV fluorescence quantification.

1.2. Keywords	Flow cytometry, extracellular vesicles, fluorescence calibration, R5 peptide, silica particles
1.3. Experiment variables	The fluorescence properties of R5-FITC peptide-labeled silica particles.
1.4. Organization name and address	Institute of Materials and Environmental Chemistry, HUN-REN Research Centre for Natural Sciences, 1117 Budapest, Magyar tudósok körútja 2., Hungary
1.5. Primary contact name and email address	Zoltán Varga (varga.zoltan@ttk.hu)
1.6. Date or time period of experiment	September 2024 – May 2026
1.7. Conclusions	The in-house synthesized silica particles were labeled with R5-FITC peptides in a simple, fast and straightforward manner. The resulted particles exhibited homogeneous fluorophore coverage while preserving their excellent colloidal stability and high monodispersity. After comprehensive characterization, FCM analysis was conducted and the MESF values of the particles were determined. Most importantly, the R5-FITC peptide-labeled silica particles were suitable for the MESF calibration of the fluorescence of antibody-labeled EVs without the need for extrapolation.
1.8. Quality control measures	CytoFLEX Ready to Use Daily QC Fluorospheres (Product No:C65719) was used for daily quality control of the used flow cytometer.
2.1.1.1. (2.1.2.1., 2.1.3.1.) Sample description	R5-FITC peptide-labeled silica particles, red blood cell-derived EVs (REVs)
2.1.1.2. Biological sample source description	Human blood
2.1.1.3. Biological sample source organism description	Human
2.1.2.2. Environmental sample location	N/A
2.3. Sample treatment description	R5-FITC peptide-labeled silica particles were purified via centrifugation. REVs were isolated from whole blood through multiple centrifugation and washing steps, followed by week-long incubation in PBS and final purification by size-exclusion chromatography.
2.4. Fluorescence reagent(s) description	Anti-CD235a-FITC (Invitrogen 11-9987-82, eBioscienceTM, 0.5 mg/mL) and matched isotype control (Invitrogen 11-4732-42, eBioscienceTM, 0.2 mg/ml).

3.1. Instrument manufacturer	Beckman Coulter, Inc.
3.2. Instrument model	CytoFLEX S
3.3. Instrument configuration and settings	The instrument was used without any custom modifications. Standard startup and quality control procedures were performed prior to measurements. For the measurement of single particles, Violet-Side Scatter (VSSC) detection was used. This VSSC channel served as the trigger, with a threshold of 3000 arbitrary units. The detector gain was set to 250 for the VSSC channel, 2000 for the FITC channel, and 1000 for the APC channel.
4.1. List-mode data files	Data files are available on request from the authors.
4.2. Compensation description	No compensation was applied.
4.3. Data transformation details	No data transformation was applied.
4.4.1. Gate description	No gating was applied because both the silica particles and the REVs are monodisperse.
4.4.2. Gate statistics	N/A
4.4.3. Gate boundaries	N/A

MIFlowCyt-EV framework

1.1 Preanalytical variables conforming to MISEV guidelines.	12 mL of a blood sample was collected from a healthy donor in K_3EDTA blood collection tubes (Greiner Bio-One) and centrifuged (800 RCF for 10 min at 4 °C). Red blood cells (RBCs) were collected, and the supernatant (platelet-rich plasma) was discarded. Next, RBCs were washed three times with 5 mL of physiological saline solution and centrifuged (800 RCF for 10 min at 4 °C) to remove residual platelets and buffy coat. Then, 5 mL of PBS (pH 7.4) was added, and RBCs were incubated at 4 °C for a week to produce EVs. This was followed by two consecutive sedimentations (2500 RCF for 10 min and 3000 RCF for 15 min at 4 °C, respectively). Next, the aliquoted supernatant was pelleted (16,000 RCF for 30 min at 4 °C), and the pellets were resuspended in 0.5 mL of PBS. A final purification step was performed using an Izon qEVoriginal (70 nm) SEC column, following the manufacturer's protocol. After loading the EV sample, elution was performed using 10 mM PBS solution (pH 7.4). The void volume was set to 2.7 mL, and 0.5 mL fractions were collected. Fraction 2, containing the

	highest concentration of REVs, was kept at 4 °C until further investigations.
1.2 Experimental design according to MIFlowCyt guidelines.	The study aimed to develop dim fluorescent silica reference particles for calibrating extracellular vesicle fluorescence. Three sizes of silica particles were synthesized and labeled with R5-FITC peptides, followed by physicochemical characterization and flow cytometric analysis. Red blood cell-derived EVs were used as biological reference samples for assessing the applicability of the particles for low-MESF fluorescence calibration.
2.1 Sample staining details	6 µL of 1.18×10^{11} particles/mL SEC-purified REV sample and 86 µL of PBS were mixed thoroughly in a 0.5 mL amber microcentrifuge tube. Next, 8 µL of anti-CD235a-FITC antibody solution was added. To validate specificity, a matched isotype control mixture was prepared: 6 µL of 1.18×10^{11} particles/mL SEC-purified REV sample and 74 µL of PBS were mixed, and 20 µL of FITC isotype control was added. Samples were incubated at room temperature for 60 min on an orbital shaker.
2.2 Sample washing details	The sample was not subjected to any washing steps after labeling.
2.3 Sample dilution details	6 µL of 1.18×10^{11} particles/mL REV was added to 8 µL of anti-CD235a-FITC and 86 µL of PBS. For FCM measurement the mixture was further diluted 215x in HEPES-NaCl buffer, resulting in a ~3.6×10^{3}-fold dilution.
3.1 Buffer alone controls.	A buffer control was measured using 0.2 µm filtered HEPES-NaCl under identical flow cytometer acquisition settings as the sample. The buffer control had a count of approximately 129 events s^{-1}. See Fig. S6.
3.2 Buffer with reagent controls.	A buffer with reagent control was analyzed using identical flow cytometer acquisition settings as for the sample. See Fig. S6.
3.3 Unstained controls.	Unstained control (REV only) was analyzed at the same dilution as their corresponding stained samples, under identical flow cytometer acquisition settings as the sample. No significant differences were observed in scatter signals, but a shift in fluorescence was observed. See Fig. 4.
3.4 Isotype controls.	Isotype control was analyzed at the same concentration and same dilution as the corresponding stained samples. Flow cytometer acquisition settings were the same, including

	triggering threshold, voltages and flow rate. No significant differences in scatter signals were observed between stained samples and their matched isotype controls. However, fluorescence intensities differed, and the isotype controls showed fluorescence levels similar to those of unstained samples. See Fig. S6.
3.5 Single-stained controls.	Single-stained controls were not included.
3.6 Procedural controls.	Procedural controls were not used in this experiment.
3.7 Serial dilutions.	No serial dilutions were performed; all samples were tested at a single concentration.
3.8. Detergent treated EV-samples	No detergent lysis experiments were performed.
4.1 Trigger Channel(s) and Threshold(s).	For the measurement of single particles, 405 nm Violet-Side Scatter (VSSC) detection was used. VSSC channel served as the trigger, with a threshold of 3000 arbitrary units. The detector gain was set to 250 for the VSSC channel, 2000 for the FITC channel, and 1000 for the APC channel.
4.2 Flow Rate / Volumetric quantification.	Each sample was analyzed for 60 seconds at a flow rate of 10 µL/min.
4.3 Fluorescence Calibration.	Arbitrary FITC fluorescence channel units were converted to MESF values using two different bead sets. Quantum FITC-5 MESF beads (Bio Rad, Cat. FCSC555A) were used for fluorescence calibration. The calibration curve was prepared based on measured median FITC-A intensities versus corresponding bead FITC MESF units provided by manufacturer. Fluorescence calibration was also performed using the developed R5-FITC-labeled silica particles. The MESF values of the R5-FITC-labeled silica particles were determined by comparing their fluorescence intensity signal to that of a solution of fluorescein of known concentration, normalized by the particle concentration determined previously via spICP-MS. The calibration curve was prepared by plotting the particles' MESF units against their MFI values.
4.4 Light Scatter Calibration.	Side-scatter intensities were calibrated to scattering cross-section values using the traceably characterized blank silica particles and Mie theory implemented in the FCMPASS software.
5.1 EV diameter/surface area/volume approximation.	REVs had a mean diameter of 179 nm with a standard deviation of 36.3 nm, determined by microfluidic resistive pulse sensing (MRPS). Because the refractive index of REVs was not

	estimated, FCM-based calculations of diameter were not performed. However, the scattering cross-section distribution was determined following calibration with the blank silica particles.
5.2 EV refractive index approximation.	The EV refractive index was not estimated.
5.3 EV epitope number approximation.	No estimation of EV epitope number was performed.
6.1 Completion of MIFlowCyt checklist.	MIFlowCyt checklist has been completed, see above.
6.2 Calibrated channel detection range	The low-MESF calibration range was 278–2053 MESF units, as determined using R5-FITC-labeled silica particles, whereas the calibration range obtained using Quantum FITC-5 MESF beads was 5000–210,000 MESF units.
6.3 EV number/concentration	Anti-CD235a-FITC antibody-labeled REVs had a concentration of 3.29×10^7 particles/mL.
6.4 EV brightness	Anti-CD235a-FITC antibody-labeled REVs had an MESF value of 390.
7.1. Sharing of data to a public repository	The datasets used and/or analyzed during the current study are available from the corresponding authors on reasonable request.
